# Feasibility and safety of transcranial direct current stimulation in the treatment of adolescent depression in a naturalistic inpatient setting: a double-blind randomized controlled trial

**DOI:** 10.1038/s41598-026-56839-1

**Published:** 2026-06-22

**Authors:** Franziska Martin, Hannah Brauer, Alexander Prehn-Kristensen, Julia Siemann, Martin Holtmann, Tanja Legenbauer, Michael Siniatchkin

**Affiliations:** 1 LWL University Hospital Hamm for Child and Adolescent Psychiatry, Ruhr University Bochum, Bochum, Germany; 2 West German Center for Child and Adolescent Health (WZKJ), Partner Site Bochum, Bochum, Germany; 3 Institute for Child and Adolescent Psychiatry, Centre for Integrative Psychiatry, School of Medicine, Christian-Albrecht University Kiel, Kiel, Germany; 4 Department of Psychology, Faculty of Human Sciences, MSH Medical School Hamburg-University of Applied Sciences and Medical University, Hamburg, Germany; 5 University Clinic of Child and Adolescent Psychiatry, Psychosomatics and Psychotherapy, RWTH Aachen University, Aachen, Germany; 6 West German Center for Child and Adolescent Health (WZKJ), Partner Site Aachen, Aachen, Germany; 7 Department of Child and Adolescent Psychiatry and Psychotherapy Bethel, Evangelical Hospital Bielefeld, Bielefeld, Germany; 8 West German Center for Child and Adolescent Health (WZKJ), Partner site Bielefeld, Bielefeld, Germany

**Keywords:** Transcranial direct current stimulation, Feasibility, Depression, Adolescents, Psychology, Depression

## Abstract

Transcranial direct current stimulation (tDCS) has potential as a treatment for adult depression, but its effectiveness in adolescents remains unexplored. This study evaluated the feasibility, safety, and efficacy of tDCS in young in-patients with depression. In a randomized, double-blind, sham-controlled trial, 34 adolescent in-patients (mean age 15.48 years) received ten sessions of either tDCS or sham stimulation over two weeks, alongside standard treatment. Depression severity, quality of life, emotional and behavioral issues, and executive functions were assessed before, immediately after, and two weeks post-treatment. The results indicated that tDCS was feasible and well-tolerated, with 28 patients completing at least eight sessions. Side effects were reported in nearly half of the sessions (45% sham, 48% tDCS), primarily mild to moderate, and no sessions were interrupted due to discomfort. Both groups experienced significant improvements in depressive symptoms and high treatment satisfaction, but no significant differences were found between the tDCS and sham groups. In conclusion, while tDCS appears to be a safe treatment option for adolescents with depression, this study could not show it to be superior to sham treatment. Further research with greater statistical power is needed to control for covariates.

**Supplementary Information:**

The online version contains supplementary material available at 10.1038/s41598-026-56839-1.

## Introduction

 Adolescent depression, affecting 5–8% of youth^1,2^, is a leading cause of disability, impacting psychosocial development, education, and employability. It is also a major risk factor for suicide, the third leading cause of death in young people worldwide^3^, and increases the risk of adult depression^4^. 

Current treatments for adolescent depression, including pharmacological and psychotherapeutic interventions, often show lower efficacy and remission rates compared to adults^5,6^. For example, the “Treatment for Adolescents With Depression Study” (TADS) found a remission rate of only around 40% with combined medication and psychotherapy^7^, a result echoed in a recent meta-analysis of adolescents with depressive episodes receiving psychotherapy^8^. Additionally, treatment adherence is often poor due to side effects^9,^ and pharmacological treatments have demonstrated only small to medium effects^10–12^.

There is an urgent need for alternative treatments. One promising option is transcranial direct current stimulation (tDCS), a non-invasive neuromodulation technique that applies low-intensity electrical currents to the brain^13^. TDCS has been shown to be safe, effective, and well tolerated, with advantages over traditional therapies, such as portability, low cost, and minimal side effects^14^. In adults, anodal stimulation targeting the left dorsolateral prefrontal cortex (lDLPFC) has shown promise as a treatment for depression, with effects comparable to those of pharmacological and psychotherapeutic interventions^15^. In large studies, anodal tDCS over the lDLPFC and cathodal tDCS over the right DLPFC (rDLPFC) significantly reduced depressive symptoms in adults^16,17^. Two recent meta-analyses of adult patients with depression showed that active tDCS was superior to sham in reducing depressive symptoms^18,19^.

Recent studies in adults, which have failed to consistently demonstrate positive effects of tDCS in depression, underscore the importance of carefully considering the specific conditions under which tDCS is administered^20,21^. Due to varying treatment durations, stimulation intensities, electrode placements, and additional therapeutic interventions, it is difficult to compare the results. No clinical trials have specifically examined tDCS for treating adolescent depression. Although previous research suggests that tDCS can have similar effects in healthy adolescents as in adults^22^, that it is safe and well tolerated^23^, there is a lack of evidence for its efficacy in treating adolescent depression. A recent literature review identified 33 studies on tDCS in adolescents with psychiatric disorders and summarized that tDCS led to a reduction of symptoms in autism spectrum disorder, ADHD, auditory hallucinations in schizophrenia, catatonia, anxiety, obsessive-compulsive disorder, and craving^24^. It also showed that tDCS was well tolerated and feasible for patients of different ages and with different psychiatric symptoms^24^. Given the promising results in other areas of child and adolescent psychiatry, tDCS could offer a new, non-pharmacological treatment for adolescent depression.

Adolescents represent a particularly vulnerable group for tDCS treatment, given the complexity of pubertal brain development. A recent study showed, for example, increasing skull thickness between childhood and early adulthood (12 to 25 years of age)^25^, a relevant factor for stimulation intensity. Another relevant factor in the study of tDCS in adolescents with psychiatric disorders would be the possible interaction of tDCS with medication. Quidé and colleagues^26^ discuss that medication addresses the limbic system (bottom-up), whereas tDCS has effects on the prefrontal cortex and hence the top-down system. In addition, concerns from adolescents and their families about the method, as well as challenges in integrating tDCS into a young person’s life, may impact both its acceptance and effectiveness.

This is the first study to examine the clinical use of tDCS in adolescents with depression. It explores its feasibility, potential barriers to treatment, and collects initial data on its effectiveness in this population. These factors will inform the design of larger, more definitive trials.

This study aims to investigate the initial efficacy and feasibility of tDCS for adolescent depression. We hypothesize that tDCS over the lDLPFC and rDLPFC, in addition to treatment as usual (TAU), will be a feasible, well-tolerated, and effective method for reducing depressive symptoms. Specifically, we expect that adolescents receiving active tDCS will report a greater reduction in depressive symptoms compared to those receiving sham stimulation.

## Results

### Descriptive statistics

The clinical and demographic characteristics of the groups are presented in Table 1. The groups did not differ significantly regarding age, IQ (Culture Fair Intelligence Test-Scale 20; CFT 20-R^27)^, severity of depression (Beck’s Depression Inventory; BDI^28^), quality of life (KIDSCREEN;^29)^, burden, and expression of emotional and behavioral issues (Strengths and Difficulties Questionnaire; SDQ^30^ and Clinical Global Impression Scale; CGI^31^), as well as executive functions (Stroop^32^ and Trail Making Test; TMT^33,34^) at PRE. In both groups, twelve patients took antidepressant medication (i.e., 66.67% within the sham group and 75.0% within the tDCS group). The number of medicated patients in the groups did not differ (*X*^2^(9, *N* = 34) = 0.283; *p* = .595; Cramer’s V = 0.132).

**Table 1:** Clinical and demographic characteristics of participants separated by tDCS verum and sham group.

	Group (n)	Mean	*SD*	df	*T*	*p*
Age (in years)	Sham (18)	15.39	1.33	32	− 0.375	0.710
	Verum (16)	15.56	1.36			
Height (in cm)	Sham (18)	168.88	6.41	32	0.375	0.71
	Verum (16)	167.84	9.53			
Weight (in kg)	Sham (18)	67.62	17.40	32	− 0.301	0.766
	Verum (16)	69.45	18.01			
IQ	Sham (18)	102.56	9.56	32	0.101	0.920
	Verum (16)	102.94	12.44			
BDI-II T1	Sham (18)	38.44	8.20	32	0.649	0.521
	Verum (16)	36.63	8.10			
KID self-report T1	Sham (17)	32.18	6.37	31	1.03	0.310
	Verum (16)	30.13	4.90			
SDQ sum self-report T1	Sham (18)	15.39	9.56	32	− 0.084	0.933
	Verum (16)	15.69	11.09			
Stroop Interference T1 (in seconds)	Sham (18)	86.31	18.79	32	− 0.736	0.467
	Verum (16)	91.38	21.35			
TMT A T1 (in seconds)	Sham (17)	22.88	9.33	31	− 0.973	0.338
	Verum (16)	25.96	8.85			
TMT B T1 (in seconds)	Sham (16)	56.04	21.81	29	− 1.261	0.218
	Verum (15)	65.35	19.11			
CGI severity T1	Sham (16)	5.44	0.73	28	0.038	0.970
	Verum(14)	5.43	0.51			

### Feasibility and side effects

Ten participants did not complete all treatment sessions: Seven in the sham group and three in the tDCS group. Among those in the sham group, one participant discontinued after one, three, four, seven, and nine sessions, respectively. Two participants completed eight sessions. In the tDCS group, one participant discontinued after four, six and nine sessions, respectively. Their reason for discontinuation was early release. One participant gave no reason for discontinuation. The data for those who completed all ten sessions can be found in the supplement.

**Fig. 1:**
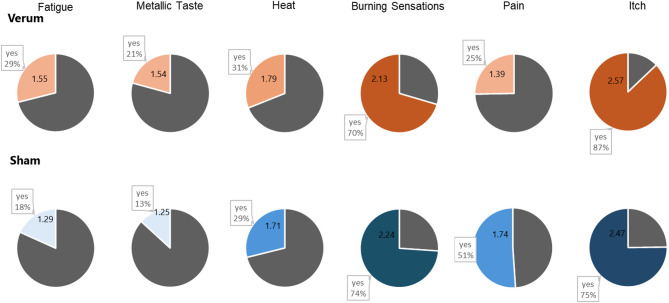
Occurrence and intensity of adverse effects during all stimulation sessions in both tDCS and sham groups. Within the charts, means of adverse effects intensity are depicted. The adverse effects were reported on a four-point Likert scale (1 = not at all; 2 = mild; 3 = moderate; 4 = strong). Adverse effects did not differ significantly between sham and tDCS groups.

Figure 1 shows the incidence and severity of side effects. Overall, they were reported in 45.48% of all sham sessions and occurred in 47.81% of all tDCS sessions. Severe AEs did not occur. All side effects were transient. None of the reported side effects was seen at a relevantly higher frequency in the tDCS than in the sham group (*U*_fatigue_ = 34.500; *Z* = -1.505; *p* = .132; *U*_metallic taste_ = 86.000, *Z* = -:980, *p* = .488; *U*_heat_ = 76.000; *Z* = − 0.430; *p* = .667; *U*_burning_ = 54.000; *Z* = − 0.396 *p* = .692; *U*_pain_ = 34.000; *Z* = -1.734 *p* = .083; *U*_itch_ = 134.000; *Z* = − 0.345 *p* = .730). Sham and tDCS stimulation did not provoke different side effects concerning their incidence and intensity.

In all sessions, the patients were unable to differentiate between tDCS and sham stimulation. They did not differ in their assumption of whether they received sham or tDCS (*X*^2^(4, *N* = 33) = 2.449; *p* = .654; Cramer’s V = 0.141). Parents could neither differentiate between sham and tDCS, and there were also no group differences (*X*^2^(5, *N* = 33) = 3.640; *p* = .602; Cramer’s V = 0.135).

Patients from both groups were similarly satisfied with the treatment after stimulation (caregivers: *t*(18) = 1.579; *p* = 132; self-report: *t*(29) = 0.400; *p* = .692). Participants reported a high level of satisfaction (mean = 2.73; SD = 0.63), which was mirrored by parent ratings (mean = 2.70; SD = 0.66).

### Efficacy of brain stimulation

**Fig. 2:**
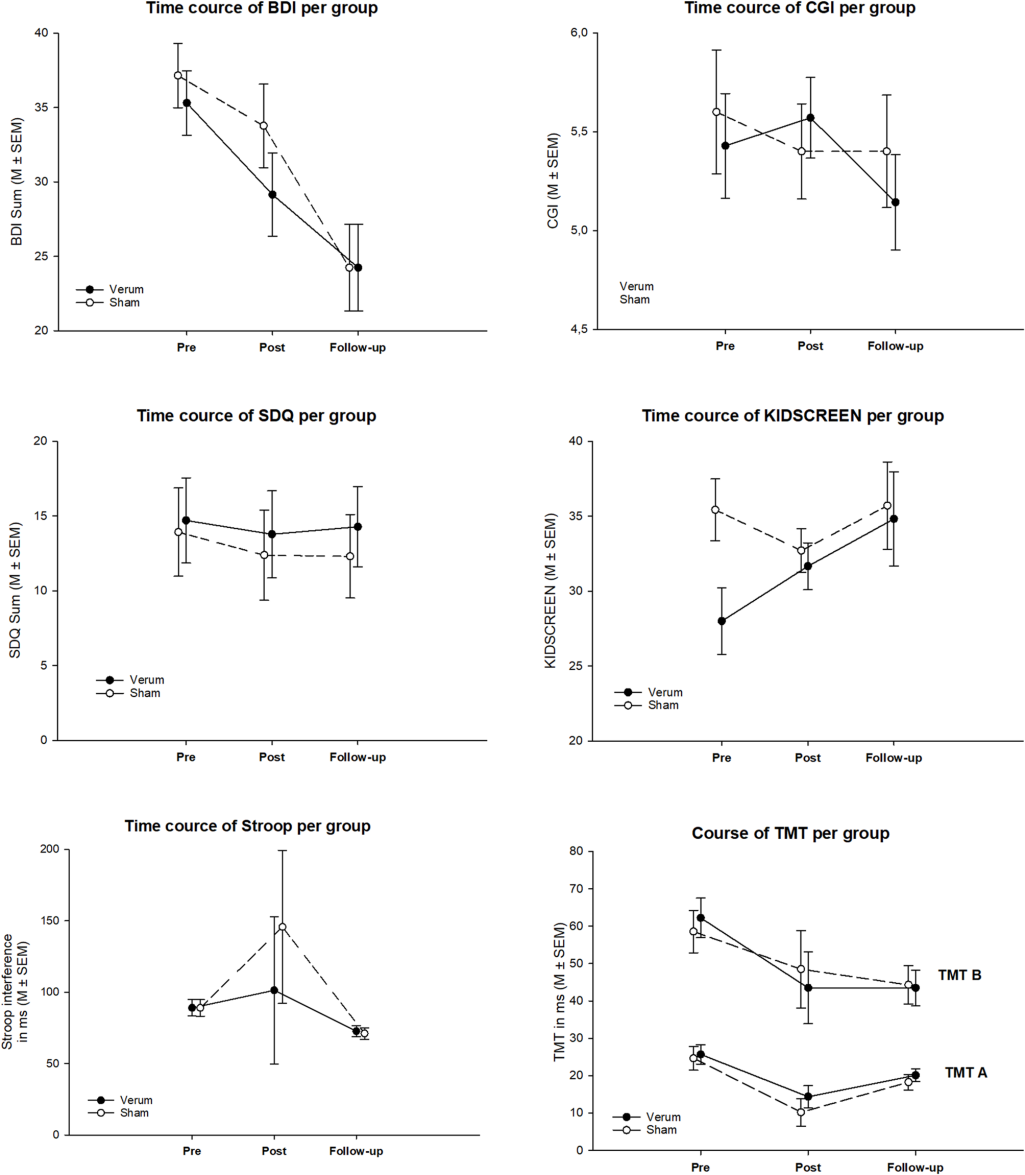
Time-courses of psychiatric symptoms and affective, behavioral and cognitive (dys) functions are depicted.

Figure 2 gives information about the time courses of the questionnaire data and Table 2 depicts results from all ANOVAs. For assessment of depression (BDI), the repeated-measures ANOVA revealed a main effect of *TIMEPOINT*. Participants reported a significant decrease of depressiveness between PRE and POST (*p* = .005; M_*Diff*_ = 4.77; 95%-CI[1.28,8.26]) and also between POST and FOLLOW UP (*p* = .003; M_*Diff*_ = 7.231; 95%-CI[2.32,12.14]) and PRE and FOLLOW UP (*p* < .001; M_*Diff*_ = 12.00; 95%-CI[7.28,16.72]).

A main effect of TIMEPOINT was found for the TMT-A, with decreasing values from PRE to POST (*p* = .003; M_*Diff*_ = 11.98; 95%-CI[3.68,20.28]) and to FOLLOW UP (*p* = .008; M_*Diff*_ = 5.58; 95%-CI[1.30,9.86]) and no differences between POST and FOLLOW UP (*p* = .065; M_*Diff*_ = -6.40; 95%-CI[-13.11,0.31]). There was a main effect TIMEPOINT for the TMT-B with decreasing values from PRE to FOLLOW UP (*p* < .001; M_*Diff*_ = 15.62; 95%-CI[7.05,24.19]) but no differences between PRE and POST (*p* = .117; M_*Diff*_ = 13.78; 95%-CI[-2.65,30.20]), and POST and FOLLOW UP (*p* > .999; M_*Diff*_ = 1.85; 95%-CI[-15.74,19.44]). After applying the Holm correction, this effect was no longer significant.

For the SDQ, there was a main effect of TIMEPOINT found for the self-report. Patients reported decreasing values from PRE to POST (*p* = .043; M_*Diff*_ = 1.234; 95%-CI[0.31,2.44]) but no difference between POST and FOLLOW UP (*p* < .999; M_*Diff*_ = -0.212; 95%-CI[-1.38,0.96]) and PRE and FOLLOW UP (*p* = .194; M_*Diff*_ = 1.022; 95%-CI[-0.34,2.38]). After applying the Holm correction, this effect was no longer significant.

**Table 2:** Repeated measures ANOVAs for clinical and neuropsychological measurements between sham and tDCS, at PRE, POST and FOLLOW-UP.

Measurement	*N*	Effect	df	F	*p*	pη^2^	pHolm
BDI	n_tDCS_ = 13 n_sham_ = 13	**TIMEPOINT**	**2**	**24.765**	**< 0.001**	**0.508**	**0.02**
		GROUP	1	0.459	0.504	0.019	1.00
		GROUP*TIMEPOINT	2	0.915	0.396	0.037	1.00
CGI severity	n_tDCS_ = 7 n_sham_ = 5	TIMEPOINT	2	0.791	0.459	0.073	1.00
		GROUP	1	0.096	0.763	0.073	1.00
		GROUP*TIMEPOINT	2	0.577	0.558	0.055	1.00
Stroop Interference	n_tDCS_ = 14 n_sham_ = 13	TIMEPOINT	2	1.600	0.218	0.060	1.00
		GROUP	1	0.791	0.459	0.011	1.00
		GROUP*TIMEPOINT	2	0.277	0.603	0.016	1.00
TMT-A	n_tDCS_ = 14 n_sham_ = 12	**TIMEPOINT**	**2**	**10.809**	**< 0.001**	**0.311**	**0.02**
		GROUP	1	0.560	0.462	0.023	1.00
		GROUP*TIMEPOINT	2	0.043	0.900	0.002	0.90
TMT-B	n_tDCS_ = 6 n_sham_ = 7	**TIMEPOINT**	**2**	**4.863**	**0.029**	**0.245**	0.55
		GROUP	1	0.132	0.721	0.009	1.00
		GROUP*TIMEPOINT	2	0.421	0.588	0.027	1.00
KIDSCREEN self	n_tDCS_ = 7 n_sham_ = 5	TIMEPOINT	2	1.445	0.259	0.116	1.00
		GROUP	1	2.554	0.138	0.188	1.00
		GROUP*TIMEPOINT	2	1.348	0.280	0.109	1.00
SDQ self	n_tDCS_ = 14 n_sham_ = 13	**TIMEPOINT**	**2**	**3.689**	**0.034**	**0.129**	0.61
		GROUP	1	0.119	0.733	0.005	1.00
		GROUP*TIMEPOINT	2	0.746	0.475	0.029	1.00

## Discussion

This randomized, double-blind, sham-controlled feasibility study investigated the use of tDCS in adolescents with depression. The key findings were: (1) tDCS was feasible, safe, and well tolerated in this population, with 95% of participants completing at least 80% of their sessions. Adverse effects (AEs) were mild to moderate, with no significant differences between tDCS and sham in terms of incidence or intensity. (2) Both tDCS and sham groups showed significant reductions in depressive symptoms from severe depressive symptoms to a moderate level and improvements in other clinical parameters, suggesting that tDCS is a safe treatment but may not offer superior benefits over sham stimulation in this context.

In the current study, tDCS appears to be highly feasible and well tolerated, consistent with previous research on children and adolescents (for review see^35^). In line with our previous studies, participants in the current study showed high acceptance and low dropout rates^36^. The technical simplicity of tDCS enhances its feasibility and patients’ compliance^23^. And the good feasibility may also be explained by a good safety and tolerability of tDCS. In accordance with other studies^35^, reported side effects were typical for tDCS—mild itching or skin irritation—which were not severe enough to cause participants to discontinue treatment. These results reinforce the idea that tDCS may be a viable treatment addition to traditional therapies, particularly for those who struggle with medication side effects or poor adherence. Whereas tDCS appears well tolerated, its efficacy in the inpatient setting, particularly when added to TAU, remains uncertain and warrants further investigation before it can be considered for routine clinical use.

Despite its feasibility, the current study was not sufficiently powered to detect potential differences between groups and therefore was unable to demonstrate the superiority of tDCS over sham stimulation in reducing depressive symptoms in this study. Both the tDCS and sham groups showed similar symptom reductions after two weeks, with a further improvement seen at the two-week follow-up. These results are inconsistent with earlier adult studies^16,17^, which reported stronger effects. Altered cortical excitability, differences in the clinical course of depression with lower response to traditional pharmacological and cognitive-behavioural treatments, compared with adults, as well as very high placebo rates, are possible factors influencing response to stimulation in young patients^37^. Brunoni et al.^16^ provided treatment for adults, whereas we studied adolescents. The effects of tDCS may vary between adults and adolescents, with adolescents potentially having different brain excitability and cortical responses to stimulation^22^. The effect of 2 mA tDCS (as in this study) has not been studied systematically. Studies have also suggested that adolescents may have a lower response to brain stimulation than adults due to differences in brain development and the clinical course of depression^38,39^.

In line with our results, a number of recent randomized, double-blind, sham-controlled multicenter clinical trial on tDCS in adults with depression^20,21,40,41^ have not been able to replicate the results of Brunoni et al.^16^. All these studies were not able to demonstrate superiority of tDCS towards sham stimulation. The authors discuss that a large placebo effect, short stimulation duration, and lack of individual stimulation intensity led to tDCS not being more effective than sham.

Although the study was underpowered to detect the intended group differences, the absence of significant between-group effects should also be interpreted in the context of confounding factors: Participants received comprehensive multimodal care, including psychotherapy, structured daily routines, and close clinical monitoring, which may have produced substantial symptom improvement across both groups. Such a treatment environment could have resulted in ceiling effects, thereby limiting the ability to detect additional benefits of the stimulation intervention. To consider this aspect, further studies are needed on patients who do not obtain such intensive concurrent therapy, for example patients in the out-patient setting, medication naïve or without additional treatment.

The electrode arrangement used in this study may not have been optimal for adolescents. The electrode arrangement used in this study is based on the insight that in major depressive disorder the left DLPFC shows hypofunction and the right DLPFC shows hyperfunction^42^. Therefore, an increase in cortical excitability through anodal stimulation at the left DLPFC and a decrease in cortical excitability through cathodal stimulation over the right DLPFC are reasonable. Whereas anodal stimulation over the left DLPFC and cathodal stimulation over the right DLPFC is commonly used in adult depression treatment, it has not consistently shown efficacy, particularly in adolescent populations^43^. The protocol was adapted from adult studies, and therefore may not fully reflect the neurodevelopmental characteristics of adolescent populations. Other electrode placements, such as anodal stimulation over the left DLPFC and cathodal stimulation over the right orbitofrontal cortex^44^, might be more effective. Although the placement of the target electrode is the same, the current flow differs and so does its influence on brain networks^44^. However, its efficacy in adolescents remains unclear. To date, there have been no studies on the effect of tDCS on depressive symptoms in adolescents. Therefore, one cannot say with certainty which electrode placement and stimulation intensity are valid for this group. However, all other montages are related to less evidence and with more risk of failure. The electrode montage in this feasibility study is based on studies of tDCS in adults with depression that have already shown an effect. In addition, based on their meta-analysis, Zhang and colleagues^18^ recommend stimulation with 2 mA to improve the antidepressant effect of tDCS. The electrode montage chosen for this study (anodal tDCS over F3 – cathodal tDCS over F4) is the most frequently used for the treatment of adult depression. This montage was suggested as the most effective and well replicated. Systematic investigation of age-appropriate stimulation parameters is therefore essential to enhance both efficacy and safety in future pediatric neuromodulation research. The sham or placebo effect is particularly effective in adolescents^45^. Therefore, studies that aim to demonstrate the effect of a treatment compared to sham treatment should have high statistical power. A waiting-control group could have improved the study design, as it would have shown, if depressive symptoms improve spontaneously and how strong the placebo effect of the sham stimulation was. Posternak and Miller^46^ found in their meta-analysis that patients show a spontaneous reduction of depressive symptoms equal to a decrease of 15.7% of the BDI score. A meta-analytic review also showed that depressive symptoms reduce spontaneously, while patients are on the waiting list^47^. Therapeutic setting, expectancy, and measurement factors influence spontaneous symptom reduction and placebo effects^48^.

Several limitations must be acknowledged. The current study is statistically underpowered for the interaction between timepoint and group. Larger studies are needed to confirm the results. Based on the preliminary findings of this study, a sample size of *N* = 86 would have been needed to find differences in depressiveness between the groups in the course of the treatment with α = 0.05 and 1-β = 0.80. Therefore, the null finding regarding the effect of tDCS on depressiveness must be interpreted with caution. A larger sample size in future studies would also be advantageous in that it would allow for analyses of subsamples. This would enable future studies to analyze more precisely who benefits from tDCS. The use of additional neurophysiological measurements would also be useful in this context. Additionally, controlling for TAU was challenging, as patients received different concurrent treatments across three clinics. This variability makes it difficult to assess the specific effect of tDCS. Future studies should standardize TAU to better isolate the effects of tDCS. Additionally, detailed data on specific classes and dosages of concomitant medication were not systematically collected. Although we recorded the number of participants per group who were taking medication, a more granular comparison of medication types between the tDCS and sham groups was not possible. Importantly, apart from antidepressants, the use of psychotropic medication constituted an exclusion criterion, which reduces potential heterogeneity related to pharmacological treatment. Nevertheless, residual confounding effects related to antidepressant use cannot be fully excluded. Although the sham condition involved only brief direct current, we cannot entirely rule out potential neurophysiological effects in the sham group^49^. It must also be acknowledged that most of the patients were medicated with antidepressants. As shown by Brunoni and others^16^, the combination of antidepressant medication and tDCS shows greater effects than tDCS or medication alone. They argue, referring to a systematic review by Quidé and colleagues^26^, that the medication addresses the limbic system (bottom-up), whereas tDCS has effects on the prefrontal cortex and hence the top-down system. Future studies should control for medication or study tDCS in drug-naïve patients.

Another limitation was the relatively restrictive inclusion criteria and the arbitrary definition of feasibility as completion of at least eight sessions. Since the use of tDCS as a therapy for children and adolescents is still a relatively new treatment option, the influence of comorbidities and various non-antidepressant medications on the effect of tDCS is difficult to predict. This would compromise the interpretability of the results. For this reason, we decided to increase the homogeneity of the sample by applying strict inclusion criteria. Furthermore, it is unclear how many tDCS sessions are required to achieve a significant clinical effect. In our recently published study with ten tDCS sessions in adolescents with ADHD, most patients were able to complete at least eight sessions^50^. The current definition of feasibility is based on these results. Recruitment exclusively from an inpatient setting may have introduced selection bias and limits the generalizability of the findings, as inpatient populations typically represent more severely affected adolescents and are treated within structured and intensive therapeutic environments. The inpatient setting was deliberately chosen for this feasibility study to ensure close clinical monitoring and safe intervention delivery in a vulnerable population. However, the results should be interpreted with caution when generalizing to broader adolescent samples, and future studies should include outpatient and multicenter recruitment to improve external validity. However, further studies are needed to investigate feasibility on an empirical basis.

Despite these limitations, the study highlights the high feasibility and tolerability of tDCS in adolescents with depression but was not sufficiently powered to detect a potential superiority of tDCS over sham stimulation in reducing depressive symptoms. Both tDCS and sham groups showed substantial improvement due to the combination of TAU and brain stimulation. Future research should explore different patient groups (e.g., out-patients with less severe symptoms), treatment conditions (e.g., drug-naïve patients), stimulation parameters (e.g., lower intensities), control conditions (e.g., waiting groups), and should include neurophysiological parameters to better understand stimulation effects.

## Methods

Data were collected at three study sites in rural and urban areas between February 2022 and May 2023. The study was approved by the university ethics committees at all sites (Ethics Committee of the Westphalia Lippe Medical Association, Ethics Committee of the Ruhr University Bochum, Ethics Committee of the Christian Albrechts University Kiel) and was conducted according to the latest version of the Declaration of Helsinki. All participants and their legal guardians gave written informed consent for participation. Their privacy rights have been observed. The study was registered at the German Clinical Trials Register (ID: DRKS00027066; date of first registration: 12/11/2021). All experiments were performed in accordance with relevant guidelines and regulations. The primary outcome measure was defined as the efficacy of brain stimulation (i.e. reduction of BDI scores from pre to post to follow-up). The secondary outcome measure was feasibility (i.e., number of completed sessions, dropout rate) and safety (i.e., reported side effects) and reduction of comorbid symptoms and impairments (i.e., SDQ scores and enhancement of neurocognitive performance in Stroop and TMT from pre to post to follow-up).

### Participants

Adolescent in-patients aged 13–17 with depression (ICD-10) and Beck Depression Inventory score > 15 (BDI; ^28^) and IQ > 80 were eligible for the study. Exclusion criteria were premature birth (before the 37th week), birth weight < 2500 g, developmental or neurological disorders, family history of epileptic seizures, history of craniocerebral injury, bipolar disorder, schizophrenia, or reported suicidality, substance consumption, and regular medication besides antidepressant drugs, body electronic devices or implants, pregnancy, scalp dermatological conditions, expected low compliance, and caregivers with insufficient German-speaking abilities. Discharge from inpatient treatment resulted in the discontinuation of tDCS sessions but did not end participation in the study.

**Fig. 3:**
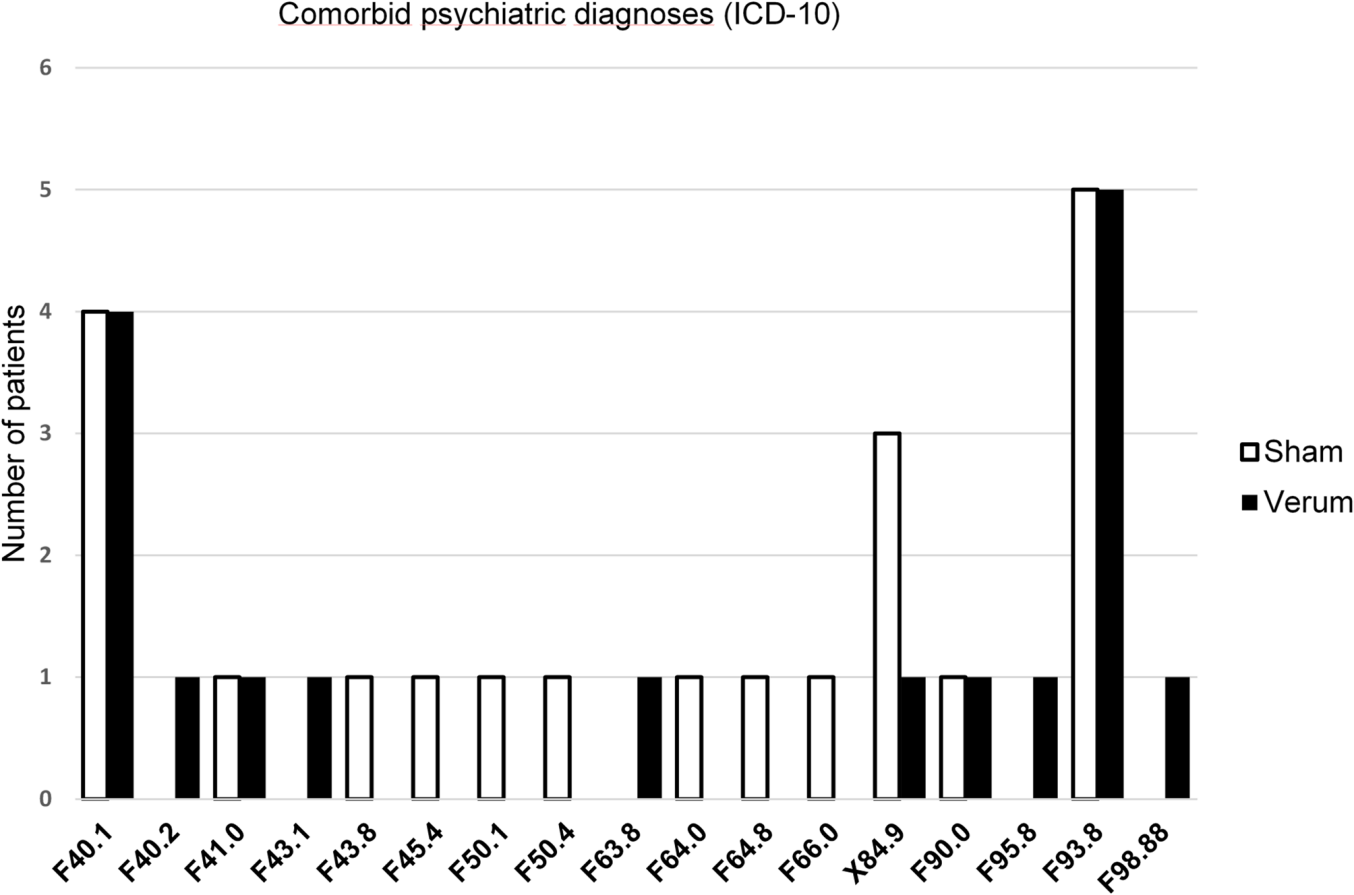
Psychiatric diagnoses (according to ICD-10) of all patients in the tDCS and sham group.

A total of 34 participants (16 in the tDCS group, 18 in the sham group) enrolled, with 12 females in each group and two non-binary patients in the sham group. Most participants reported comorbid psychiatric conditions, as summarized in Fig. 3.

### Study design

This randomized, double-blind, sham-controlled feasibility study investigated the effects of tDCS in adolescent in-patients receiving TAU. TAU comprised cognitive behavioral therapy, nursing care, psychomotor, and art therapy, medication. Antidepressant medication comprised serotonin-reuptake inhibitors. Therapy was in accordance with the guidelines on depression in children and adolescents. After meeting inclusion and exclusion criteria, participants and caregivers provided informed consent. They were randomly assigned to either the tDCS or sham group, with gender-balanced randomization. Randomization was performed automatically by the stimulation device. An individual participation number was assigned to each patient upon enrolment. This number was linked to randomization into one of the two stimulation groups before the start of the study by the company providing the devices. This ensured blinding of the staff, patients, and caregivers.

Before stimulation (PRE), participants completed assessments on depression, comorbidities, quality of life, IQ, and executive functions. Caregivers also completed questionnaires on quality of life and expectations (caregiver data can be found in the supplement). Over the following two weeks, participants underwent ten sessions of tDCS or sham stimulation on a daily basis, engaging in low-level cognitive activities (i.e., talking with the study staff, reading, drawing). Side effects were documented, and any AEs were reported to attending physicians and, if severe, to the ethical committees for possible termination.

Post-stimulation (POST, two weeks after PRE), participants and caregivers repeated the assessments. A follow-up assessment (FOLLOW UP) took place two weeks later with the same measurements, including treatment satisfaction and assumptions about the type of stimulation received. Throughout the study, all participants received TAU, which included standard care for adolescent depression in Germany: nursing, medical and psychological care, cognitive-behavioral therapy, antidepressant medication, parent counseling, and group therapy. No participants were involved in other studies or used other treatments for depression or comorbidities.

### Transcranial direct current stimulation

Direct current was applied through a pair of saline-soaked surface electrodes (35cm^2^) and delivered by a battery-driven, CE-certified constant current stimulator (neuroConn Co., Illmenau, Germany). Anodal tDCS was applied over the lDLPFC (F3 according to the International 10–20 system) and cathodal tDCS over the rDLPFC (F4) for 30 min with 2 mA and a current density of 0.80 A/m^2^. During the tDCS condition, the current was ramped up and down to and from 2 mA over 8 s. During the sham condition, the current was ramped up for 8 s, followed by 15 s of 2 mA stimulation; then it was ramped down for 8 s. The impedance was controlled by the device throughout each tDCS session, kept at < 10 kΩ and limited by the voltage.

### Assessments

#### Feasibility and tolerability

The feasibility was assessed by comparing the number of sessions with tDCS or sham stimulation completed overall and in each group. Moreover, the stimulation was considered feasible, if the patient was able to complete at least eight sessions of stimulation. And finally, the drop-out rate was determined. For assessment of tolerability/side effects and blinding effectiveness, a standardized safety questionnaire^51,52^ was used. Participants rated the incidence and intensity of the six most common tDCS side effects (itching, pain, burning sensations, heat, metallic taste, fatigue) on a four-point Likert-scale (0 = not experienced to 3 = strongly experienced). The time period between the last and the current tDCS or sham session was addressed. It was documented when the sensation occurred, how long it lasted or whether it still lasted and whether action against it was undertaken.

#### Beck’s depression inventory revision

Participants filled in the Beck Depression Inventory Revised (BDI^28^), a self-report questionnaire assessing the degree of depressive symptoms within the last 14 days. It consists of 21 items representing depressive symptoms with at least four answer options with increasing intensity (0 to 3). In this study, the BDI was applied at three timepoints (PRE, POST, and FOLLOW UP) and showed high reliability (Cronbach’s alpha = 0.819).

#### Strengths and difficulties questionnaire

Parents and participants filled out the Strengths and Difficulties Questionnaire (SDQ^30^), a standardized self-report questionnaire with 25 items assessing behavioral problems during the past six months. Participants rate, whether the descriptions of behaviors apply to them on a three-point Likert-scale (“not true”, “somewhat true”, “certainly true”). Reliability in the current sample was high (Cronbach’s alpha = 0.779). The SDQ was also filled in by the participants’ caregiver (see supplement). The items are identical to the self-report (Cronbach’s alpha = 0.675).

#### KIDSCREEN-10

The KIDSCREEN-10 ^29^ assesses health-related quality of life in children and adolescents during the last week. It contains ten items, which can be rated on a five-point Likert-scale between 1 (“not at all” / “never”) and 5 (“extremely” / “always”). An additional item asks about the general impression of their health (“excellent”, “very good”, “good”, “fair”, “poor”). The current sample showed acceptable reliability (Cronbach’s alpha = 0.603). Caregivers also filled in the KIDSCREEN-10 (see supplement; Cronbach’s alpha = 0.713), which contains the same items as the self-report.

#### Clinical global impression scale (CGI)

The Clinical Global Impression Scale (CGI^31^) was filled in by the participants’ therapists. This instrument assesses the severity of a mental illness and the overall improvement on a 7-point Likert scale (“normal/not at all ill”, “very much improved” to “among the most extremely ill patients”, “very much worse”). It also provides an efficacy index, which indicates the efficacy of a treatment depending on the severity of side effects.

#### Satisfaction with treatment and expectation questionnaire

Caregivers’ and participants’ expectations towards the treatment were assessed at PRE. The questionnaire comprised questions about the reasons for participation, the expected improvement of depressive symptoms through the treatment, questions about anticipated side effects and why they consented to participate. They answered on a 7-point Likert-scale (1 = “does not apply” to 7 = “fully applies”).

Participants and caregivers were furthermore asked about their satisfaction with the treatment at POST. They could answer on a 4-point Likert scale.

#### Stroop task

The Stroop colour and word test^32^ measures the ability to inhibit cognitive interference and processing speed. Participants are presented with three tables and are asked to read out loud colour words printed in black, name the colour of coloured lines or name the colour of words printed in interfering colours. Each table was presented in three versions. The time was recorded for each matrix. The median was calculated per table.

#### Trail making test

The Trail Making Test (TMT^33,34^) is a paper-pencil test used to measure deficits in attention and executive dysfunctions. It consists of two tasks, A and B. Participants are prompted to connect numbers from one to 25 as fast as possible in task A. Time is recorded and errors are noted. In part B, participants are asked to connect numbers from one to 13 and letters from A to K in alternating order (1 – A – 2 – B etc.). Time is stopped and errors are noted.

### Statistics

Group differences concerning demographic, clinical, and psychological characteristics were calculated with two-tailed independent t-tests. Group differences in AEs were assessed using a Mann-Whitney U Test. A repeated-measures ANOVA was calculated with all dependent variables (BDI, CGI, SDQ_parents_ and SDQ_patients_, Stroop, TMT) with *TIMEPOINT* (PRE, POST and FOLLOW UP) as a within-subject factor and *GROUP* (sham vs. tDCS) as a between-subject factor. Greenhouse-Geisser correction was applied. Post-hoc t-tests were Bonferroni corrected. To control for multiple testing across the seven instruments, Holm–Bonferroni correction was applied to the *p*-values of each effect. The significance level was set at *p* < .05.

## Supplementary Information

Below is the link to the electronic supplementary material.

Supplementary Material 1

Supplementary Material 2

## Data Availability

The data that support the findings of this study are not openly available due to reasons of sensitivity and are available from the corresponding author upon reasonable request.
