# Supplementary material for: Feasibility and safety of transcranial direct current stimulation in the treatment of adolescent depression in a naturalistic inpatient setting: a double-blind randomized controlled trial
Source: Sci Rep. 2026 Jun 22;16:19338. doi: 10.1038/s41598-026-56839-1 (PMC13287699; doi:10.1038/s41598-026-56839-1)
Supplement: Supplementary file 1 — Supplementary Material 1 [file 41598_2026_56839_MOESM1_ESM.docx]

**Table 1 supplement.** *Clinical characteristics externally assessed by the caregivers separated by tDCS verum and sham group.*

|  | **Group (n)** | ***Mean*** | ***SD*** | **df** | ***T*** | ***p*** |
| --- | --- | --- | --- | --- | --- | --- |
|  | Verum(16) | 30.13 | 4.90 |  |  |  |
| KID caregiver T1 | Sham (18) | 28.72 | 7.30 | 32 | .320 | .759 |
|  | Verum(16) | 28.06 | 4.65 |  |  |  |
|  | Verum(16) | 15.69 | 11.09 |  |  |  |
| SDQ sum caregiver T1 | Sham (18) | 14.31 | 7.48 | 27 | -0.219 | .828 |
|  | Verum(16) | 15.08 | 11.24 |  |  |  |

**Table 2 supplement.** *Repeated measures ANOVAs for caregiver data between sham and tDCS, at PRE, POST and FOLLOW-UP.*

| Measurement | N | Effect | df | F | p | pη^2^ |
| --- | --- | --- | --- | --- | --- | --- |
| KIDSCREEN caregiver | n_tDCS_ = 7  n_sham_ = 7 | TIMEPOINT | 2 | 1.124 | .339 | .086 |
|  |  | GROUP | 1 | 3.127 | .102 | .207 |
|  |  | GROUP*TIMEPOINT | 2 | 0.674 | .512 | .053 |
|  | n_tDCS_ = 3  n_sham_ = 6 | TIMEPOINT | 2 | 2.308 | .149 | .248 |
| SDQ caregiver |  | GROUP | 1 | 0.032 | .863 | .005 |
|  |  | GROUP*TIMEPOINT | 2 | 0.206 | .774 | .029 |
