# Supplementary material for: Feasibility and safety of transcranial direct current stimulation in the treatment of adolescent depression in a naturalistic inpatient setting: a double-blind randomized controlled trial
Source: Sci Rep. 2026 Jun 22;16:19338. doi: 10.1038/s41598-026-56839-1 (PMC13287699; doi:10.1038/s41598-026-56839-1)
Supplement: Supplementary file 2 — Supplementary Material 2 [file 41598_2026_56839_MOESM2_ESM.docx]

**Table 1** *Repeated measures ANOVAs for clinical and neuropsychological measurements between sham and tDCS, at PRE, POST and FOLLOW-UP, only completers (i.e., completed ten sessions of tDCS) are shown*

| Measurement | N | Effect | df | F | p | pη^2^ | pHolm |
| --- | --- | --- | --- | --- | --- | --- | --- |
| BDI | n_tDCS_ = 11  n_sham_ = 10 | **TIMEPOINT** | **2** | **16.926** | **< .001** | **.471** | **0.021** |
|  |  | GROUP | 1 | 1.030 | .323 | .051 | 1.000 |
|  |  | GROUP*TIMEPOINT | 2 | 0.777 | .452 | .038 | 1.000 |
| CGI severity | n_tDCS_ = 6  n_sham_ = 4 | TIMEPOINT | 2 | 0.756 | .486 | .086 | 1.000 |
|  |  | GROUP | 1 | 0.139 | .719 | .017 | 1.000 |
|  |  | GROUP*TIMEPOINT | 2 | 0.577 | .575 | .064 | 1.000 |
| Stroop Interference | n_tDCS_ = 12  n_sham_ = 11 | TIMEPOINT | 2 | 1.630 | .208 | .072 | 1.000 |
|  |  | GROUP | 1 | 0.331 | .571 | .016 | 1.000 |
|  |  | GROUP*TIMEPOINT | 2 | 0.392 | .538 | .018 | 1.000 |
| TMT-A | n_tDCS_ = 12  n_sham_ = 10 | **TIMEPOINT** | **2** | **8.160** | **< .001** | **.290** | **0.021** |
|  |  | GROUP | 1 | 0.009 | .924 | < .001 | 1.000 |
|  |  | GROUP*TIMEPOINT | 2 | 0.006 | .975 | < .001 | 0.975 |
| TMT-B | n_tDCS_ = 5  n_sham_ = 5 | **TIMEPOINT** | **2** | **4.855** | **.018** | **.306** | 0.342 |
|  |  | GROUP | 1 | 0.059 | .813 | .005 | 1.000 |
|  |  | GROUP*TIMEPOINT | 2 | 1.102 | .339 | .091 | 1.000 |
| KIDSCREEN self | n_tDCS_ = 7  n_sham_ = 5 | TIMEPOINT | 2 | 2.401 | .122 | .231 | 1.000 |
|  |  | GROUP | 1 | 0.940 | .361 | .105 | 1.000 |
|  |  | GROUP*TIMEPOINT | 2 | 2.026 | .192 | .202 | 1.000 |
| SDQ self | n_tDCS_ = 9  n_sham_ = 10 | TIMEPOINT | 2 | 0.194 | .823 | .011 | 1.000 |
|  |  | GROUP | 1 | 0.311 | .584 | .018 | 1.000 |
|  |  | GROUP*TIMEPOINT | 2 | 1.094 | .346 | .060 | 1.000 |
